# Fermented Milk as an Alternative Therapy for Obesity

**DOI:** 10.1155/ijfo/5584818

**Published:** 2026-07-20

**Authors:** Ulan Safitri, Ninik Rustanti, Adriyan Pramono, Suparmi Suparmi

**Affiliations:** ^1^ Department of Nutrition Science, Universitas Diponegoro, Semarang, Indonesia, undip.ac.id; ^2^ Undergraduate Nutrition Study Program, Sekolah Tinggi Ilmu Kesehatan Bakti Nusantara Gorontalo, Gorontalo, Indonesia; ^3^ Department of Biology, Universitas Islam Sultan Agung, Semarang, Indonesia, unissula.ac.id

**Keywords:** fatty liver, fermented milk, lipid metabolism, obesity, probiotics, SREBP-1c

## Abstract

Obesity is a global health concern closely associated with metabolic disorders, including fatty liver disease. Conventional treatment approaches rely on lifestyle modifications and pharmacological interventions, but functional foods, particularly fermented milk, have gained attention as promising alternatives. Fermented milk containing probiotics has emerged as a potential functional food for managing obesity and its complications, including nonalcoholic fatty liver disease (NAFLD). This review summarises current evidence on the role of probiotic‐fermented milk in modulating lipid metabolism and related molecular pathways, with a focus on Sterol Regulatory Element‐Binding Protein‐1c (SREBP‐1c). Probiotic fermentation can improve the composition of the gut microbiota, increase short‐chain fatty acid production and regulate bile acid metabolism, thereby decreasing hepatic lipogenesis. Probiotic strains, such as *Lactobacillus fermentum* and *Lactobacillus rhamnosus*, have been reported in preclinical studies to downregulate SREBP‐1c expression through multiple mechanisms, including activation of AMP‐activated protein kinase (AMPK), short‐chain fatty acid–mediated GPR43 signalling and modulation of bile acid pathways (FXR/FGF15). In addition, probiotic‐fermented milk modulates systemic immunity through the gut–spleen–liver axis, thereby reducing chronic, low‐grade inflammation associated with NAFLD. Recent in vivo and limited clinical studies have shown that probiotic‐fermented milk reduces SREBP‐1c expression, decreases hepatic triglyceride accumulation and improves insulin sensitivity. Collectively, these findings suggest that probiotic‐fermented milk may be an effective dietary strategy for obesity‐related liver disorders. Further clinical studies are needed to validate the therapeutic potential and optimise formulations for targeted interventions.

## 1. Introduction

Obesity has risen substantially in recent decades. The World Health Organisation reports that global obesity prevalence has tripled since 1975, affecting more than 650 million adults in 2021 and markedly increasing the risk of Type 2 diabetes, cardiovascular disease and nonalcoholic fatty liver disease (NAFLD) [1]. NAFLD, characterised by excessive hepatic fat accumulation in the absence of clinically significant alcohol consumption, can progress to nonalcoholic steatohepatitis (NASH), fibrosis and cirrhosis. Its prevalence rises in parallel with obesity [2]. The pathogenesis of obesity‐related fatty liver is multifactorial, driven by chronic positive energy balance, dysregulated lipid handling, insulin resistance and low‐grade systemic inflammation [3, 4]. Given the limited long‐term efficacy of conventional approaches such as lifestyle modification and available pharmacotherapies, interest has grown in complementary strategies, including functional foods, to prevent or ameliorate obesity‐associated hepatic disease.

Probiotic‐fermented milk, including products such as yoghourt and kefir, has emerged as a promising functional food. Fermented dairy can modulate gut microbial communities, alter short‐chain fatty acid (SCFA) production and deliver bioactive peptides and conjugated linoleic acid (CLA). These effects have been associated with favourable shifts in lipid metabolism and reductions in hepatic steatosis [5, 6]. A central molecular nexus in lipid homeostasis is Sterol Regulatory Element‐Binding Protein‐1c (SREBP‐1c), the master transcription factor that drives hepatic de novo lipogenesis. SREBP‐1c is upregulated in obesity and contributes directly to triglyceride accumulation and NAFLD progression [7]. Preclinical studies indicate that specific probiotic strains—such as *Lactobacillus fermentum* and *Lactobacillus rhamnosus*—can attenuate SREBP‐1c expression via multiple mechanisms [8, 9], making SREBP‐1c a plausible mechanistic target through which fermented milk may exert metabolic benefits.

Although prior reviews have addressed aspects of fermented dairy′s antiobesity potential, important gaps remain. Manzanarez‐Quín et al. [10] reviewed probiotic strains and bioactive peptides but did not evaluate SREBP‐1c as a unifying molecular target or synthesise strain‐specific signalling pathways. Earlier syntheses, such as that by Pothuraju et al. [11], predate much of the mechanistic literature that has since emerged on SCFA–GPR43 signalling, AMP‐activated protein kinase (AMPK)–mediated suppression of SREBP‐1c and bile acid–FXR/FGF15 crosstalk [12]. These developments invite an integrated appraisal that links microbial, metabolic and host signalling pathways.

This review addresses three interrelated novelties. First, it consolidates current evidence through the specific lens of SREBP‐1c regulation, integrating AMPK activation, SCFA‐mediated GPR43 signalling and bile acid–FXR/FGF15 modulation into a cohesive mechanistic framework. Second, it highlights the gut–spleen–liver immunometabolic axis as an additional, underexplored pathway through which probiotic‐fermented milk may attenuate obesity‐associated hepatic inflammation. Third, it provides a structured, criterion‐based synthesis of preclinical and clinical studies (Table 1), distinguishing animal and human evidence and identifying methodological limitations that constrain translational interpretation. By evaluating the literature within this integrated mechanistic context, the review is aimed at clarifying the therapeutic potential of probiotic‐fermented milk for obesity‐related hepatic disorders and delineating priorities for future research.

**Table 1:** Preclinical (animal) studies on the effects of fermented milk on obesity and lipid metabolism.

Authors/year	Population	Methodology	Intervention	Main findings
G. Refaat et al. [13]	35 rats (28 obese)	4 groups: HFD only, HFD + fermented milk (Rayeb), HFD + vitamin D, HFD + Rayeb + vitamin D	Fermented milk (Rayeb), vitamin D or their combination	Fermented milk reduced body weight, fat percentage and improved liver/kidney function and lipid profile
Al‐Sheraji et al. [14]	Sprague–Dawley rats	Randomised controlled study, 4 groups: Standard diet, high‐cholesterol diet and high‐cholesterol + fermented milk with *B. longum* BB536	Fermented milk with *B. longum* BB536	Improved expression of LDL receptor and reduced expression of HMG‐CoA reductase in hypercholesterolemic rats
Alhamid & Mousawi [15]	28 male rats	4 groups: Standard diet, HFD, HFD + yoghourt + aloe vera gel, HFD + fermented milk with *B. lactis* BB‐12 + aloe vera gel	Synbiotic‐fermented milk + aloe vera gel	Fermented milk + aloe vera reduced body weight, improved lipid profiles and decreased liver weight in HFD rats
Aljutaily et al. [16]	48 obese Wistar rats	Divided into 6 groups:	16‐h fasting, fermented camel milk (with or without Sukari dates) or their combination	The changes in predominance from Bacteroidota to Firmicutes after treatments indicate a possible rise in short‐chain fatty acid synthesis. Analysis of the functional pathway predicted increased activity in carbohydrate degradation pathways
		• Control group		
		• Intermittent fasting group		
		• Fermented camel milk group		
		• Intermittent		
		• Fasting + fermented camel milk		
		• Fermented came		
		• l milk with Sukari dates		
		Intermittent fasting + fermented camel milk with Sukari dates		
Chen et al. [8]	Rats with NAFLD	Controlled feeding study	*Lactobacillus rhamnosus* hsryfm 1301 fermented milk	Regulated lipid metabolism and reduced inflammation, with beneficial effects on NAFLD‐related parameters
Rani et al. [17]	High‐fat diet‐induced obese mice	Controlled feeding study	Probiotic‐ and prebiotic‐fermented milk (*Lactobacillus fermentum* NCDC 400 + fructo‐oligosaccharide)	Reduced body weight, improved lipid profile and decreased hepatic steatosis
Makwana et al. [18]	High‐fat diet‐induced obese Wistar rats	Controlled feeding study	Probiotic‐fermented milk	Reduced body weight, epididymal fat mass and blood glucose levels
Dahiya et al. [19]	Mice	Controlled feeding study	Conjugated linoleic acid–enriched skim milk fermented with *Lactobacillus fermentum* DDHI27	Improved lipid profile and positively altered transcription of adipogenesis‐ and lipogenesis‐related genes; demonstrated antiobesity effects
Pothuraju et al. [11]	High‐fat diet‐fed C57BL/6 J mice	Controlled feeding study	Milk fermented with *Lactobacillus plantarum* NCDC 625, alone or in combination with herbs	Decreased body weight and fat mass, attributed to upregulation of UCP‐2 expression and reduced proinflammatory cytokines

## 2. Fermented Milk and Probiotics in Modulating SREBP‐1c‐Mediated Lipogenesis: Mechanisms and Evidence

### 2.1. Obesity and Fatty Liver Risk

Obesity is a major risk factor for NAFLD, with visceral adiposity playing a crucial role in its development and progression [20]. Excess visceral fat contributes to liver fat accumulation by releasing free fatty acids (FFAs), inflammatory cytokines and adipokines [21]. This leads to systemic inflammation and insulin resistance, creating a vicious cycle that exacerbates hepatic steatosis [21]. Insulin resistance, a common feature in obesity, promotes increased lipogenesis and impairs the liver′s ability to metabolise fat effectively [22]. Additionally, adipose tissue dysfunction in obesity is characterised by increased fat deposition and chronic low‐grade inflammation. This condition further contributes to the pathogenesis of NAFLD. Understanding the complex interplay between adipose tissue and the liver in obesity is essential for developing new preventive and therapeutic approaches for NAFLD management [23].

**Figure 1:**
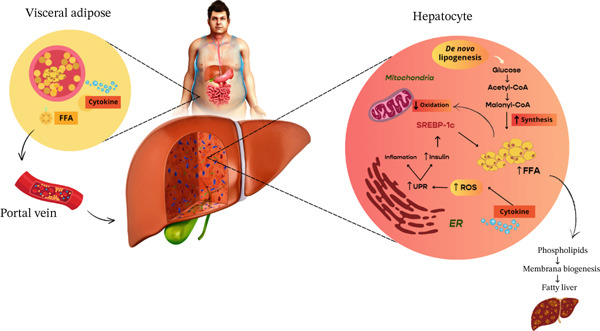
Fatty liver risk in obesity. This is an original figure created by the authors using Canva (Canva Pty Ltd.).

Figure 1 depicts the risk of fatty liver in obesity. Visceral adiposity releases FFAs and proinflammatory cytokines into the liver. The high FFA influx into the liver leads to fat accumulation in hepatocytes. Additionally, proinflammatory cytokines released from adipose tissue increase insulin resistance in liver cells. This insulin resistance leads to increased activity of the transcription factor SREBP‐1c, which triggers de novo lipogenesis—the formation of new fat in the liver. In this process, glucose is converted into acetyl‐CoA and then into malonyl‐CoA, which is used to synthesise fatty acids and triglycerides. Triglyceride accumulation in the liver is accompanied by impaired mitochondrial fatty acid oxidation, thereby increasing oxidative stress through the generation of *reactive oxygen species* (ROS). ROSs induce a stress response in the *endoplasmic reticulum* (ER), which helps maintain cellular homeostasis through the *unfolded protein response* (UPR). However, if stress persists, this response leads to increased inflammation and worsens insulin resistance, forming a cycle that aggravates the condition. The combination of fat accumulation, elevated ROS and chronic inflammation leads to liver cell damage. It increases the risk of NAFLD, which can progress to NASH, fibrosis or even cirrhosis at more advanced stages.

Research indicates that SREBP‐1c plays a crucial role in hepatic lipogenesis and is regulated by insulin‐dependent and independent mechanisms [24]. While feeding can induce SREBP‐1c expression through insulin‐independent pathways, obesity‐related induction is primarily insulin‐dependent [25]. In obesity and Type 2 diabetes, chronic hyperinsulinemia led to paradoxical activation of SREBP‐1c despite insulin resistance in muscle and adipose tissues, exacerbating hepatic lipogenesis [24]. Carbohydrate‐responsive element‐binding protein (ChREBP) also contributes to lipogenesis regulation, activated by glucose independently of insulin [26]. Given SREBP‐1c′s central role in lipid metabolism, targeting its pathway is a potential strategy for treating metabolic diseases such as Type 2 diabetes, insulin resistance and fatty liver [27].

Biological mechanisms contributing to NAFLD′s growth and progression involve the interactions between lipid metabolism, insulin resistance, inflammation and oxidative stress. Insulin resistance plays a central role, leading to increased hepatic glucose production and reduced fat oxidation, exacerbating liver steatosis [28]. Lipotoxicity promotes inflammation and insulin resistance, creating a vicious cycle that accelerates NAFLD progression [29]. Oxidative stress, resulting from mitochondrial dysfunction and increased ROS production, contributes to hepatocellular damage and disease progression [30]. While chronic inflammation in adipose tissue is associated with insulin resistance [31], surprisingly, suppressing adipocyte inflammation can exacerbate insulin resistance and impair adipose tissue function [32]. Inflammatory cytokines, adipokines and growth factors mediate adipogenesis and insulin resistance in obesity [3].

Dysfunctional visceral fat, characterised by inflammation, fibrosis and impaired angiogenesis, contributes to systemic insulin resistance and metabolic syndrome [33]. Adipokines and cytokines, including tumour necrosis factor‐alpha (TNF‐*α*), IL‐6, adiponectin and leptin, play crucial roles in these processes. Proteomic studies have identified key regulators of NF‐*κ*B pathways involved in inflammation and insulin resistance [31]. The interplay between these factors leads to hepatocyte injury, inflammation and fibrosis, driving NAFLD progression from simple steatosis to more severe forms like NASH and cirrhosis [28].

### 2.2. SREBP‐1c and Lipid Metabolism

**Figure 2:**
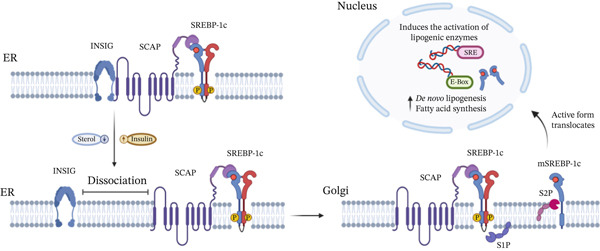
The activation of Sterol Regulatory Element‐Binding Protein‐1c (SREBP‐1c). This is an original figure created by the authors using BioRender (BioRender.com).

SREBP‐1c is a crucial transcription factor regulating lipid metabolism in the liver. It controls gene expression in fatty acid, triglyceride and cholesterol synthesis. SREBP‐1c activation is primarily mediated by insulin and ER stress [7]. SREBP‐1c is synthesised as an inactive precursor in the ER and requires proteolytic activation. The *SREBP cleavage–activating protein* (SCAP) is essential for SREBP activation, and both have emerged as potential therapeutic targets in cancer treatment [34]. In insulin‐resistant states, ER stress can activate SREBP‐1c independently of insulin, contributing to hepatic steatosis [7, 35]. Various signalling kinases and phosphorylation events regulate its activity. SREBP‐1c works with other transcription factors, particularly ChREBP, to coordinate postprandial glycolysis and lipogenesis. While SREBP‐1c primarily mediates insulin′s effects on lipogenic genes, ChREBP responds to glucose, inducing both glycolytic and lipogenic genes [36]. The SREBP pathway also involves insulin signalling, innate immunity and cancer development [37]. The activation of SREBP‐1c is shown in Figure 2.

Beyond its role in lipogenesis, SREBP‐1c has been identified as a key regulator of fatty acid synthesis and desaturation pathways. SREBP‐1c directly upregulates the expression of acetyl‐CoA carboxylase (ACC) and fatty acid synthase (FAS) enzymes. These enzymes are essential for the conversion of acetyl‐CoA to malonyl‐CoA and, subsequently, to long‐chain fatty acids [38]. Additionally, SREBP‐1c regulates the activity of Stearoyl‐CoA Desaturase‐1 (SCD1), an enzyme responsible for converting saturated fatty acids into monounsaturated fatty acids, which are critical for triglyceride storage and membrane fluidity [39]. Dysregulation of SREBP‐1c, particularly its overactivation, can lead to excessive lipid accumulation, promoting hepatic steatosis and metabolic dysfunction.

SREBP‐1c is highly sensitive to nutritional status and hormonal regulation. Insulin is the primary activator of SREBP‐1c, stimulating its transcription through the PI3K/Akt/mTORC1 signalling pathway [26]. This mechanism ensures that lipogenesis is upregulated in response to postprandial surges in glucose and insulin. Conversely, fasting and glucagon signalling suppress SREBP‐1c expression, thereby reducing lipogenesis and promoting fatty acid oxidation. Additionally, omega‐3 fatty acids have been shown to downregulate SREBP‐1c activity by inhibiting its nuclear translocation, providing a potential dietary strategy to mitigate hepatic lipid accumulation [26].

Excessive activation of SREBP‐1c is closely linked to the development of metabolic disorders such as NAFLD, insulin resistance and Type 2 diabetes. In obesity, chronic hyperinsulinemia sustains SREBP‐1c activity, driving continuous lipid synthesis despite systemic insulin resistance [25]. This paradoxical effect contributes to hepatic lipid overload and inflammation, accelerating the progression from simple steatosis to NASH [40]. Furthermore, elevated SREBP‐1c expression in adipose tissue has been implicated in adipocyte hypertrophy and dysfunction, exacerbating systemic insulin resistance and dyslipidaemia [40].

Given its central role in lipid metabolism, SREBP‐1c represents an attractive therapeutic target. Small‐molecule inhibitors of SREBP‐1c processing, such as fatostatin, have been explored for their ability to reduce hepatic lipogenesis and improve insulin sensitivity. Additionally, dietary interventions, including polyphenol‐rich foods and omega‐3 fatty acids, have shown potential to modulate SREBP‐1c activity and ameliorate metabolic dysfunction [41]. Future research should focus on elucidating the precise regulatory networks governing SREBP‐1c activity and identifying novel therapeutic strategies for metabolic diseases linked to lipid dysregulation.

The activation of SREBP‐1c involves its dissociation from insulin‐induced gene (INSIG) and transport from the ER to the Golgi, facilitated by SCAP [42]. In the Golgi, SREBP‐1c undergoes proteolytic cleavage by S1P and S2P, releasing its transcriptionally active N‐terminal domain, which then translocates to the nucleus to promote lipogenesis [43]. Various factors, including ammonia from glutamine metabolism, stimulate this process [44]. Notably, ER stress can activate Caspase‐2, which cleaves S1P to generate an active fragment that initiates SCAP‐independent SREBP activation, potentially contributing to steatohepatitis [45]. The dysregulation of this pathway can lead to excessive lipogenesis and increase the risk of fatty liver disease, making the SCAP/SREBP axis a potential therapeutic target for metabolic disorders [42].

Recent studies suggest that SREBP‐1c is intricately connected to other metabolic pathways beyond lipid synthesis. One such interaction involves its regulation by peroxisome proliferator–activated receptors (PPARs), a family of nuclear receptors that govern lipid metabolism and energy homeostasis. PPAR*α* activation has been shown to inhibit SREBP‐1c expression, reducing hepatic lipogenesis while promoting fatty acid oxidation [26]. Conversely, PPAR*γ*, which is primarily involved in adipogenesis, has been implicated in increasing SREBP‐1c activity, further linking adipose tissue expansion to hepatic lipid accumulation.

Additionally, SREBP‐1c has been found to interact with Fibroblast Growth Factor 21 (FGF21), a key metabolic regulator produced in the liver. FGF21 has been shown to suppress SREBP‐1c‐mediated lipogenesis while enhancing lipid oxidation and ketogenesis, particularly under fasting conditions [46, 47]. Understanding how FGF21 signalling influences SREBP‐1c activity could open new avenues for therapeutic interventions in metabolic disorders.

Chronic activation of SREBP‐1c contributes not only to excessive lipid accumulation but also to cellular stress and lipotoxicity. The excessive synthesis of fatty acids and triglycerides can lead to ER stress, mitochondrial dysfunction and oxidative stress. All of those conditions contribute to the progression of metabolic diseases [48]. Studies have shown that SREBP‐1c‐induced lipotoxicity can impair mitochondrial function by increasing the accumulation of ceramides and diacylglycerol, leading to insulin resistance and hepatocellular damage [49].

Furthermore, evidence suggests that the interplay between SREBP‐1c and autophagy is crucial in maintaining lipid homeostasis. Autophagy, a cellular process responsible for degrading and recycling cellular components [50], is negatively regulated by SREBP‐1c, contributing to lipid droplet accumulation in hepatocytes [51]. Modulating autophagy through pharmacological or dietary interventions may represent a therapeutic strategy to mitigate SREBP‐1c‐induced metabolic dysfunction.

SREBP‐1c activation has also been linked to hepatic inflammation and fibrosis, key features in the progression from NAFLD to NASH. The upregulation of lipogenic genes by SREBP‐1c is often accompanied by increased expression of proinflammatory cytokines such as TNF‐*α* and Interleukin‐1*β* (IL‐1*β*), leading to hepatic inflammation [50]. Additionally, lipid overload caused by SREBP‐1c activation can induce hepatic stellate cell activation, promoting extracellular matrix deposition and fibrosis [52, 53].

### 2.3. Mechanisms Underlying the Antiobesity Potential of Fermented Milk

Probiotic‐fermented milk exerts its antiobesity and hepatoprotective effects through several interconnected mechanisms, spanning gut microbiota modulation, strain‐specific regulation of hepatic SREBP‐1c, bioactive peptide and CLA action and immune‐mediated pathways linking the gut to the liver.

A central mechanism is the modulation of gut microbiota composition. Probiotics such as *Lactobacillus* and *Bifidobacterium* species increase the abundance of beneficial gut bacteria while suppressing pathogenic taxa, thereby enhancing the production of SCFAs, principally acetate, propionate and butyrate [54]. SCFAs act on G protein–coupled receptors, such as GPR43, to regulate appetite‐related hormone secretion, improve insulin sensitivity, reduce systemic inflammation and promote fatty acid oxidation over lipogenesis, collectively reducing hepatic fat accumulation [55]. This microbiota–SCFA axis represents the upstream mechanism through which several of the strain‐specific effects described below converge on hepatic lipid metabolism.

At the molecular level, several probiotic strains have been shown to attenuate hepatic SREBP‐1c expression through distinct, strain‐dependent pathways. In mice fed a high‐fat diet, *Limosilactobacillus fermentum* strains MG4231 and MG4244 reduced hepatic SREBP‐1c and FAS expression via AMPK phosphorylation, independently of direct SCFA involvement [56]. In contrast, *L. fermentum* E15 in zebrafish models exerted lipid‐lowering effects through SCFA (isovaleric acid)–mediated GPR43 signalling [57]. *Lactobacillus rhamnosus* strains have been reported to reduce hepatic lipogenic and inflammatory gene expression by modulating bile acid metabolism via the FXR/FGF15 axis and upregulation of CYP7A1 [58]. *Lactobacillus johnsonii* JNU3402 alleviates hepatic steatosis through a lactate‐PKA‐SREBP‐1c pathway [9]. *Bifidobacterium animalis* subsp. *Lactis* has been shown to suppress fat accumulation via SCFA‐mediated activation of GPR43, primarily by influencing energy expenditure rather than directly targeting SREBP‐1c [59]. *Bifidobacterium bifidum* enhances hepatic mitochondrial *β*‐oxidation via the PPAR*α*/PGC‐1*α*/CPT1A pathway [60]. Acetic acid produced by other *Lactobacillus* and *Bifidobacterium* strains has additionally been linked to lipid regulation through LXR*α*–SREBP‐1 signalling [57, 61]. Collectively, these findings indicate that the SREBP‐1c‐modulating effect of fermented milk is strain‐specific and operates through at least three convergent pathways: AMPK activation, SCFA–GPR43 signalling and bile acid–FXR/FGF15 modulation—though most evidence to date derives from animal models ([9]; Schmidt et al. [62]).

Beyond probiotic cells themselves, fermentation generates bioactive peptides and CLA, both of which independently contribute to lipid regulation. During fermentation, lactic acid bacteria hydrolyse milk proteins into peptides, including caseinophosphopeptides and lactoferrin‐derived fragments. These peptides inhibit pancreatic lipase activity, thereby reducing dietary fat absorption and postprandial triglyceride levels [63], and also suppress preadipocyte differentiation and promote lipolysis [64]. CLA, whose concentration increases during fermentation via bacterial biohydrogenation of polyunsaturated fatty acids [65], modulates the activities of lipoprotein lipase and hormone‐sensitive lipase, thereby reducing fat accumulation and increasing lean body mass. Together with the bioactive peptides described above, CLA downregulates SREBP‐1c‐driven lipogenesis, providing a complementary, nonmicrobial mechanism that operates alongside the probiotic strain‐specific pathways discussed earlier.

Finally, probiotic‐fermented milk modulates systemic immunity and appetite signalling in ways relevant to obesity‐related hepatic inflammation. In a murine model of protein–energy malnutrition, probiotic‐fermented milk increased splenic IgA^+^ cells, macrophages and dendritic cells and enhanced Th1‐associated cytokine production and phagocytic activity [66]. Probiotic interaction with gut‐associated lymphoid tissue increases IL‐10 and suppresses TNF‐*α*, shifting the immune response towards a balanced Th1/Th2 profile [67].

Through the liver–spleen axis, splenic immunomodulation may help attenuate the chronic low‐grade inflammation associated with obesity‐related NAFLD [68]. In parallel, fermented milk consumption increases circulating levels of satiety hormones Glucagon‐Like Peptide‐1 (GLP‐1) and peptide YY (PYY), contributing to reduced food intake [69]. Emerging evidence also points to dairy‐derived extracellular vesicles, which carry microRNAs that influence adipogenesis and insulin sensitivity, as a potential additional avenue of action [70]. Given their early‐stage, exploratory nature, this avenue is discussed further in Section 2.6 (Future Research Directions) rather than here.

### 2.4. Evidence Synthesis: Studies on the Effects of Fermented Milk on Obesity and Lipid Metabolism

Studies included in this evidence synthesis were selected according to the following criteria: (i) The intervention consisted of fermented milk, fermented dairy products or probiotic/synbiotic‐supplemented milk or yoghourt. Studies evaluating isolated phytochemicals or nondairy compounds without a fermented milk/dairy matrix were excluded; (ii) outcomes included measures of body weight or adiposity, hepatic lipid accumulation/steatosis, circulating lipid profile (e.g. triglycerides and cholesterol) or expression of lipid metabolism–related genes/proteins (e.g. SREBP‐1c, FAS, LDL receptor and HMG‐CoA reductase); and (iii) studies were original preclinical (in vivo animal) or clinical (human) investigations published in peer‐reviewed journals. On this basis, Saleh Al‐maamari et al.′s study [71], which evaluated quercetin supplementation rather than a fermented milk intervention, was excluded. Eligible studies are presented separately for preclinical animal models (Table 1) and human/clinical studies (Table 2), reflecting their differing levels of translational evidence.

**Table 2:** Human/clinical studies on the effects of fermented milk on obesity and lipid metabolism.

Authors/year	Population	Methodology	Intervention	Main findings
Sandby et al. [72]	100 males (30–70 years) with abdominal obesity	Randomised controlled trial with 4 intervention groups; 16‐week intervention with MRI to assess liver fat	400 g/day of milk, yoghourt, heat‐treated yoghourt or acidified milk	Fermented yoghourt did not significantly reduce liver fat or improve metabolic markers compared to nonfermented milk; modest improvements in blood pressure, insulin and cholesterol across all groups
Pimentel et al. [73]	14 healthy men	Randomised crossover study with single‐dose and 2‐week daily consumption of milk or yoghourt	Fermented milk vs. yoghourt	Fermented milk modulated serum metabolites related to amino acids, bile acids and indole derivatives, suggesting a metabolic footprint that influences endogenous activity in healthy men
Bakhshimoghaddam et al. [74]	102 NAFLD patients	24‐week randomised controlled trial, 3 groups: Synbiotic yoghourt, conventional yoghourt and control	Synbiotic yoghourt	Significant reduction in liver steatosis and decreased liver enzyme levels in the synbiotic yoghourt group

The preclinical studies summarised in Table 1 collectively demonstrate that fermented milk and probiotic‐fermented dairy products consistently improve body weight, adiposity and lipid profiles across diverse rodent models of obesity and NAFLD. Several studies provide gene‐ or protein‐level evidence of altered lipid metabolism: Dahiya and Puniya [19] reported changes in adipogenesis‐ and lipogenesis‐related gene transcription following CLA‐enriched fermented skim milk, while Al‐Sheraji et al. [14] demonstrated increased LDL receptor and reduced HMG‐CoA reductase expression with *B. longum* BB536–fermented milk, although neither study directly assessed SREBP‐1c or hepatic steatosis. Pothuraju et al. [11], Rani et al. [17] and Makwana et al. [18] consistently report reductions in body weight and adiposity with probiotic‐fermented milk in high‐fat diet‐induced rodents, lending phenotypic support to the strain‐specific molecular mechanisms discussed in Section 2.3, while D. Chen et al. [8] directly link *L. rhamnosus*–fermented milk to improvements in NAFLD‐related lipid and inflammatory parameters. Notably, few of these fermented milk studies report hepatic SREBP‐1c expression directly, in contrast to the mechanistic studies of probiotic strains discussed in Section 2.3; this represents a methodological gap between phenotypic outcomes and the molecular target central to this review.

Among the human/clinical studies (Table 2), evidence remains more limited and mixed. Sandby et al. [72] found that, despite modest improvements in blood pressure, insulin and cholesterol across all dairy groups, fermented yoghourt did not significantly reduce liver fat compared with nonfermented milk over 16 weeks, suggesting that fermentation status alone may be insufficient to alter hepatic fat without additional formulation differences such as added probiotics or prebiotics. In contrast, Bakhshimoghaddam et al. [74] found that synbiotic yoghourt significantly reduced liver steatosis and liver enzyme levels in patients with NAFLD over 24 weeks, indicating that the addition of specific probiotic/prebiotic combinations rather than fermentation per se may be the more relevant determinant of hepatic benefit. Pimentel et al. [73] provide complementary mechanistic insight, showing that fermented milk alters the serum metabolome (amino acids, bile acids and indole derivatives) in healthy men, although this study did not directly assess obesity‐ or NAFLD‐related outcomes.

Taken together, the preclinical evidence in Table 1 consistently supports a role for probiotic‐fermented milk in improving lipid metabolism and reducing hepatic fat accumulation, with mechanistic plausibility reinforced by the strain‐specific SREBP‐1c pathways discussed in Section 2.3. However, the human/clinical evidence in Table 2 remains limited in volume and inconsistent in outcome, and to date, no clinical study has directly measured hepatic SREBP‐1c expression in response to fermented milk consumption. This gap between mechanistic preclinical evidence and clinical validation represents a key limitation of the current evidence base and a priority for future research, as further discussed in Section 2.6.

### 2.5. Challenges and Limitations in the Use of Fermented Milk

Implementing fermented milk products as a mainstream intervention for obesity and fatty liver disease remains fraught with several challenges and limitations. One major limitation is the variability in probiotic strains and fermentation processes, which can significantly impact the bioavailability and efficacy of bioactive compounds. Different strains of *Lactobacillus* and *Bifidobacterium* exert varying effects on lipid metabolism, and further research is needed to identify the most effective combinations.

Another challenge is consumer acceptance and adherence to consuming fermented dairy. While yoghourt and kefir are widely consumed, cultural and dietary preferences may limit their adoption in certain populations. Alternative formulations, such as plant‐based fermented milk, may be explored to enhance inclusivity and adherence. Additionally, taste preferences, texture and perceived health benefits influence consumer choices, necessitating tailored marketing strategies and consumer education programmes to promote awareness of the metabolic advantages of fermented milk products.

Additionally, the safety of long‐term probiotic consumption, especially in immunocompromised individuals, remains a concern. Although probiotics are generally regarded as safe, excessive intake or improper formulation could lead to dysbiosis or adverse metabolic effects. Standardised regulations and quality control measures are essential to ensure consistency and efficacy in probiotic‐rich dairy products. The lack of standardised clinical guidelines on probiotic dosages further complicates their integration into mainstream dietary recommendations.

Economic factors also pose a significant limitation. High production costs, specialised storage requirements and short shelf life can make fermented milk products less accessible, particularly in low‐income populations. Enhancing cost‐effectiveness through improved production methods, strain selection and alternative dairy sources could support wider adoption. Moreover, policy interventions, subsidies and public health initiatives may be required to integrate fermented milk into national dietary programmes, ensuring affordability and accessibility for diverse populations.

Finally, interactions among fermented milk, other dietary components and pharmacological treatments require further exploration. Certain probiotic strains may interact with medications or other dietary elements, influencing their efficacy. More clinical studies are necessary to assess the synergistic or antagonistic effects of fermented milk when consumed alongside other therapeutic interventions for obesity and metabolic disorders.

### 2.6. Future Research Directions

Although various studies have demonstrated the potential of fermented milk in managing obesity and the risk of fatty liver, several research areas require further exploration. The first specific area, including the exploration of strain‐specific probiotics, demands further investigation. Strain‐specific probiotic research is needed to identify the most effective microorganisms in suppressing SREBP‐1c expression and improving lipid metabolism. Strains such as *L. johnsonii* JNU3402 have shown potential to reduce liver steatosis via the lactate‐PKA‐SREBP‐1c pathway [9], but human clinical studies remain limited.

Identifying effective microorganisms through strain‐specific research is crucial. This raises a significant research gap: the need for a deeper understanding of the specific roles of individual probiotic strains in metabolic regulation. While various *Lactobacillus* and *Bifidobacterium* strains have been studied, their distinct effects on lipid metabolism remain underexplored. Future research should focus on strain‐specific interactions with host lipid metabolism, particularly regarding hepatic SREBP‐1c signalling. Furthermore, multistrain probiotic formulations should be evaluated to determine whether strain combinations provide synergistic benefits over single‐strain interventions. The development of synbiotic formulations that incorporate both probiotics and prebiotics also holds promise for enhancing metabolic outcomes [75].

The second area for harnessing the potential of fermented milk in obesity management is optimising probiotic dosages and fermentation parameters. It is critical to maximise the production of bioactive compounds such as CLA and SCFAs, which are pivotal in lipid regulation and appetite control. Additionally, long‐term clinical trials in patients with lipid dysregulation, such as NAFLD and MAFLD, are necessary to evaluate the impact of fermented milk consumption on parameters such as insulin resistance, SREBP‐1c gene expression and inflammatory biomarkers. These trials could address the responses to the diverse probiotic supplementation. Moreover, research comparing fermented milk products containing probiotics, synbiotics, parabiotics and postbiotics is needed to identify more stable and safer alternatives for individuals with specific health conditions.

Long‐term clinical trials on fermented milk and its benefits for metabolic health are needed, as most existing studies are either short‐term or conducted in animal models. Longitudinal human trials with diverse populations are necessary to evaluate the sustained effects of fermented milk on lipid metabolism, insulin sensitivity and hepatic steatosis. Understanding individual responses in these trials can also inform precision nutrition strategies, tailoring fermented milk interventions to achieve optimal metabolic benefits. Additionally, precision nutrition approaches that integrate genetic, microbiome and metabolic profiling could help personalise fermented milk interventions. For example, individuals with distinct gut microbiota compositions may respond differently to probiotic supplementation, necessitating tailored dietary strategies for optimal metabolic benefits [76].

To maximise therapeutic efficacy, future studies should explore how fermented milk consumption interacts with pharmacological treatments for obesity and fatty liver disease. For instance, the combination of probiotics with lipid‐lowering drugs such as statins or SREBP‐1c inhibitors could be investigated for potential synergistic effects. Additionally, integrating fermented milk into structured dietary and physical activity programmes could enhance weight management outcomes. Recent evidence suggests that high‐protein yoghourt fortified with prebiotics and vitamins enhances fat loss when combined with a calorie‐restricted diet [12], highlighting the need for comprehensive lifestyle‐based interventions.

Furthermore, integrating fermented milk with lifestyle interventions, such as healthy diets and exercise, may yield more significant outcomes in managing obesity and the risk of fatty liver. For instance, yoghourt fortified with nutrients such as whey protein and prebiotics has been shown to significantly reduce body fat mass when combined with calorie‐restricted diets [12]. Future studies could also explore the potential of *next-generation probiotics* (NGPs), such as *Akkermansia muciniphila*, in fermented milk products, which may offer additional benefits by improving gut microbiota composition and reducing liver inflammation [77].

Finally, omics‐based approaches, including genomics, proteomics and metabolomics, can provide deeper insights into the molecular mechanisms underlying fermented milk′s effects on lipid metabolism. By identifying specific changes in gene expression linked to SREBP‐1c regulation and SCAP/SREBP interactions, researchers can refine probiotic formulations to achieve targeted metabolic benefits [42]. Additionally, systems biology approaches can help unravel complex host–microbiome interactions, paving the way for precision‐targeted functional foods in obesity management.

By focusing on these research areas, fermented milk can further develop as an effective food‐based therapeutic approach for managing obesity and its metabolic complications. Expanding clinical research, refining probiotic formulations and integrating dietary interventions with pharmacological therapies will be key to unlocking the full potential of fermented milk in metabolic health.

## 3. Conclusion

Fermented milk products represent a promising alternative therapeutic strategy for managing obesity and preventing fatty liver disease. Its composite bioactive profile, bioactive peptides, CLA and SCFAs play a pivotal role in regulating lipid metabolism, suppressing SREBP‐1c expression and modulating the gut microbiota, collectively mitigating the risk of obesity‐related hepatic complications. Clinical and preclinical studies demonstrate their ability to reduce hepatic steatosis, improve insulin sensitivity and lower systemic inflammation. Despite these promising results, further research is needed to optimise strain‐specific probiotic formulations, determine effective dosages and explore long‐term clinical efficacy in diverse populations. Additionally, integrating fermented milk with lifestyle interventions, such as balanced diets and exercise, could enhance therapeutic outcomes. As a functional food, fermented milk offers a sustainable, accessible and practical approach to improving metabolic health and addressing global challenges posed by obesity and fatty liver disease.

**Nomenclature**
SREBP‐1c: Sterol Regulatory Element‐Binding Protein‐1c (a transcription factor regulating de novo lipogenesis and fatty acid synthesis)
NAFLD: nonalcoholic fatty liver disease (characterised by excess fat accumulation in the liver without significant alcohol consumption)
MAFLD: metabolically associated fatty liver disease (a broader classification emphasising metabolic dysfunction)
CLA: conjugated linoleic acid (a fatty acid with antiobesity properties found in fermented milk)
LAB: lactic acid bacteria (microorganisms used in milk fermentation to produce probiotics and bioactive compounds)
FFA: free fatty acid (released from adipose tissue and contributing to hepatic steatosis)
HFD: high‐fat diet (a dietary model used in studies on obesity and metabolic disorders)
ROS: reactive oxygen species (molecules that can cause oxidative stress and cellular damage)
SCFA: short‐chain fatty acid (metabolites produced by gut microbiota fermentation, influencing energy metabolism and lipid profiles)
PPAR*γ*
: peroxisome proliferator–activated receptor *γ* (a nuclear receptor involved in lipid metabolism and energy balance)
LDL: low‐density lipoprotein (often referred to as ‘bad cholesterol’, is associated with cardiovascular risks)
HDL: high‐density lipoprotein (known as ‘good cholesterol’, is involved in reverse cholesterol transport)
*Lactobacillus*
: a genus of probiotic bacteria commonly found in fermented dairy products
*Bifidobacterium*
: a genus of probiotic bacteria with beneficial effects on gut health and metabolism
IC_50_
: half‐maximal inhibitory concentration (a measure of a substance′s effectiveness in inhibiting a biological process)
ACC: acetyl‐CoA carboxylase (an enzyme involved in fatty acid synthesis)
FAS: fatty acid synthase (an enzyme catalysing fatty acid synthesis)

## Author Contributions

Ulan Safitri: conceptualisation, methodology, investigation, visualisation, and writing—original draft, review, and editing. Ninik Rustanti: conceptualisation, supervision, resources, project administration, and writing—review and editing. Adriyan Pramono: supervision, validation, and writing—review and editing. Suparmi Suparmi: resources, supervision, and writing—review and editing.

## Funding

No funding was received for this manuscript.

## Conflicts of Interest

The authors declare no conflicts of interest.

## Data Availability

The data that support the findings of this study are available on request from the corresponding author. The data are not publicly available due to privacy or ethical restrictions.
